# Associations of early pregnancy BMI with adverse pregnancy outcomes and infant neurocognitive development

**DOI:** 10.1038/s41598-021-83430-7

**Published:** 2021-02-15

**Authors:** Yu-Ting Chen, Ting Zhang, Chang Chen, Yin-Yin Xia, Ting-Li Han, Xu-Yang Chen, Xiao-Ling He, Ge Xu, Zhen Zou, Hong-Bo Qi, Hua Zhang, Benjamin B. Albert, John Colombo, Philip N. Baker

**Affiliations:** 1 School of Public Health and Management, Chongqing Medical University, Chongqing, 400016 China; 2 Department of Obstetrics and Gynaecology, The First Affiliated Hospital of Chongqing Medical University, Chongqing, 400016 China; 3 Institute of Life Sciences, Chongqing Medical University, Chongqing, 400016 China; 4 Liggins Institute, University of Auckland, Auckland, New Zealand; 5 A Better Start – National Science Challenge, Auckland, New Zealand; 6 Department of Psychology and Schiefelbusch Institute for Life Span Studies, University of Kansas, Lawrence, USA; 7 College of Life Sciences, University of Leicester, Leicester, UK

**Keywords:** Health care, Risk factors

## Abstract

The prevalence of overweight and obesity amongst reproductive women has been increasing worldwide. Our aim was to compare pregnancy outcomes and infant neurocognitive development by different BMI classifications and investigate whether early pregnancy BMI was associated with risks of adverse outcomes in a Southwest Chinese population. We analysed data from 1273 women enrolled in the Complex Lipids in Mothers and Babies (CLIMB) randomized controlled trial in Chongqing, China. Maternal BMI was classified as underweight, normal weight and overweight/obese according to the Chinese, WHO Asian, and WHO European standards. For the adverse pregnancy outcomes, after adjustment for potential confounders, an underweight BMI was associated with increased risk of small for gestational age (SGA) babies, and an overweight/obese BMI was associated with increased risk of maternal gestational diabetes mellitus (GDM), caesarean section (C-section), macrosomia and large for gestational age (LGA) babies. For infant neurocognitive development, 1017 mothers and their children participated; no significant differences were seen in the Mental Development Index (MDI) or the Psychomotor Development Index (PDI) between the three BMI groups. Our findings demonstrate that abnormal early pregnancy BMI were associated with increased risks of adverse pregnancy outcomes in Chinese women, while early pregnancy BMI had no significant influence on the infant neurocognitive development at 12 months of age.

## Introduction

Overweight and obesity is a major public health problem among women of reproductive age, with increasing worldwide prevalence. In the United States, the incidence of overweight (BMI ≥ 25 kg/m^2^) and obesity (BMI ≥ 30 kg/m^2^) among women aged 18–44 years was 20.0–33.0% and 14.5–36.4%, respectively^1^. In China, overweight (BMI ≥ 24 kg/m^2^) and obesity (BMI ≥ 28 kg/m^2^) are less common, affecting 10.8–18.2% and 1.9–6.0% of pregnant women, respectively^2–4^. The definitions of overweight and obesity from different ethnic groups or countries are diverse^5^; in China the three commonly used classifications are the Chinese, WHO Asian, and WHO European BMI classifications^6^. However, there is little evidence to determine which of the different definitions of overweight/obesity are most appropriate for Chinese populations.

It is well-established that maternal overweight and obesity are associated with several adverse pregnancy and neonatal outcomes in both mothers and their babies based on large and extensive evidence including many cohort studies and meta-analyses^2,3,6–12^. For the mother, there are increased risks of developing gestational diabetes mellitus (GDM) and delivering by caesarean section (C-section)^2,3,7,8,10^. For the baby, maternal overweight and obesity are associated with macrosomia and large for gestational age (LGA)^2,3,9^. Furthermore, obesity during pregnancy influences the subsequent health of both the mother and her offspring^13–15^, women and their children are at increased risk of cardiovascular and metabolic related diseases^15,16^. Additionally, offspring exposed to maternal obesity in utero are at risk of a range of aberrant neurodevelopmental development^17–20^, including attention deficit hyperactivity disorder (ADHD), autism spectrum disorder (ASD), developmental delay, emotional/behavioural problems, reduced general cognitive ability and nonverbal ability scores, impaired full and verbal intelligence quotient (IQ) scores and reduced Bayley’s mental development index (MDI) and psychomotor development index (PDI) scores. This may be caused by the inflammatory uterine environment that accompanies an obese state before and during pregnancy, which leads to a cascading series of events that might affect brain development and subsequent neurodevelopmental functioning^19,20^. However, the mechanisms underlying the potential effects of maternal overweight and obesity on adverse pregnancy outcomes and infant neurodevelopment are still unclear.

The aim of this study was to examine the association between early pregnancy BMI and pregnancy outcomes and infant neurocognitive development in Chinese pregnant women, and to compare the use of the different BMI cut-off points for Chinese populations. We hypothesized that abnormal maternal BMI, such as overweight/obese is associated with increased risks of adverse pregnancy outcomes and may reduce infant neurocognitive development.

## Results

### Participant characteristics

The characteristics of participants are shown in Table 1. The median maternal age was 28 years, mean early pregnancy BMI was 21.5 ± 2.9 kg/m^2^, mean GWG was 9.1 ± 3.6 kg, mean MDI was 94.6 ± 17.6 and mean PDI was 87.0 ± 14.6. Of the participating women, 98.7% were Han ethnicity and 77.5% were primiparous. Approximately 215 (16.9%), 314 (24.7%) and 142 (11.2%) of the women were overweight/obese based on the Chinese, WHO Asian and WHO European BMI cut-off points, respectively.

**Table 1:** Characteristics of the study participants.

**Characteristic**	
**Maternal characteristics**	
Maternal age (years, median (IQR))	28 (26, 31)
BMI (kg/m^2^, mean ± SD)	21.5 ± 2.9
GWG (kg, mean ± SD)^a^	9.1 ± 3.6
Han ethnicity (%)	98.7
Marital status (%)	97.9
Primiparity (%)	77.5
History of miscarriage or abortion (%)	47.3
Smoking or drinking during pregnancy (%)	0.4
**Chinese BMI category (%)**	
Underweight	11.9
Normal weight	71.2
Overweight	14.0
Obese	2.9
**Asian BMI category (%)**	
Underweight	11.9
Normal weight	63.4
Overweight	21.1
Obese	3.6
**WHO BMI category (%)**	
Underweight	11.9
Normal weight	76.9
Overweight	9.9
Obese	1.3
**Neonatal characteristics**	
Gestational age at delivery (week, mean ± SD)	39.4 ± 1.5
Birth weight (g, mean ± SD)^b^	3310.7 ± 436.9
Birth length (cm, mean ± SD)^c^	49.7 ± 1.9
Apgar score at 1 min (median (IQR))	10 (9, 10)
Apgar score at 5 min (median (IQR))	10 (10, 10)
**New born sex (n, %)**^**d**^	
Male	663 (52.2)
Female	608 (47.8)
**Infant characteristics**	
BSID test^e^	
MDI (mean ± SD)	94.6 ± 17.6
PDI (mean ± SD)	87.0 ± 14.6

*BMI* body mass index, *GWG* gestational weight gain, *BSID* Bayley Scales of Infant Development.

Values are mean ± SD, median (IQR) or n (%).

Missing data: ^a^77 last maternal weight, ^b^11 birth weight, ^c^30 birth length, ^d^2 new born sex, ^e^256 BSID tests (total n = 1017).

The complex lipid supplementation of the CLIMB trial had no effects on either pregnancy outcomes or neurocognitive development (Albert et al., unpublished data); the cohort was thus analysed as a whole rather by intervention subgroup.

### Associations between pregnancy outcomes and early pregnancy BMI by different BMI classifications

Adjusted *OR* and 95% *CI* for pregnancy outcomes in association with BMI classified using the Chinese, WHO Asian, and WHO European BMI cut-off points are shown in Table 2. After adjustment for potential confounders, early pregnancy BMI (as determined using three BMI classifications) was significantly associated with GDM, macrosomia, LGA and SGA. Namely, compared to normal weight women, 1) women who were overweight/obese had higher risk of GDM (Chinese BMI category: *OR* = 1.80, 95% *CI*: 1.29 to 2.51; WHO Asian BMI category: *OR* = 1.47, 95% *CI*: 1.09 to 1.99; WHO European BMI category: *OR* = 1.81, 95% *CI*: 1.23 to 2.68), macrosomia (Chinese BMI category: *OR* = 3.08, 95% *CI*: 1.70 to 5.57; WHO Asian BMI category: *OR* = 2.96, 95% *CI*: 1.71 to 5.12; WHO European BMI category: *OR* = 3.17, 95% *CI*: 1.60 to 6.25) and LGA (Chinese BMI category: *OR* = 2.76, 95% *CI*: 1.75 to 4.37; WHO Asian BMI category: *OR* = 2.58, 95% *CI*: 1.68 to 3.96; WHO European BMI category: *OR* = 2.52, 95% *CI*: 1.50 to 4.24); 2) women who were underweight were more likely to have pregnancies complicated by SGA (Chinese BMI category: *OR* = 2.08, 95% *CI*: 1.17 to 3.70; WHO Asian BMI category: *OR* = 2.04, 95% *CI*: 1.14 to 3.66; WHO European BMI category: *OR* = 2.10, 95% *CI*: 1.18 to 3.72). Meanwhile, overweight/obese women were at increased risk of C-section based on the Chinese and WHO Asian standard, but not the WHO European standard (Chinese BMI category: *OR* = 1.49, 95% *CI*: 1.07 to 2.06; WHO Asian BMI category *OR* = 1.40, 95% *CI*: 1.05 to 1.86, WHO European BMI category: *OR* = 1.26, 95% *CI*: 0.86 to 1.85).

**Table 2:** Adjusted *OR* (95% *CI*) for pregnancy outcomes by maternal BMI according to the Chinese, WHO Asian or WHO European classifications.

Pregnancy outcomes	BMI	Adjusted *OR* (95% *CI*)		
		Chinese BMI category	WHO Asian BMI category	WHO European BMI category
GDM	Underweight	0.67 (0.41, 1.08)	0.66 (0.41, 1.08)	0.64 (0.40, 1.04)
	Normal weight	1 (reference)	1 (reference)	1 (reference)
	Overweight/Obese	1.80 (1.29, 2.51)***	1.47 (1.09, 1.99)*	1.81 (1.23, 2.68)**
PROM	Underweight	1.49 (1.00, 2.20)*	1.42 (0.96, 2.11)	1.51 (1.02, 2.23)*
	Normal weight	1 (reference)	1 (reference)	1 (reference)
	Overweight/Obese	0.91 (0.63, 1.33)	0.77 (0.55, 1.09)	0.97 (0.63, 1.50)
C-section	Underweight	0.75 (0.50, 1.12)	0.76 (0.51, 1.14)	0.71 (0.48, 1.07)
	Normal weight	1 (reference)	1 (reference)	1 (reference)
	Overweight/Obese	1.49 (1.07, 2.06)*	1.40 (1.05, 1.86)*	1.26 (0.86, 1.85)
PTB	Underweight	0.57 (0.17, 1.88)	0.62 (0.18, 2.08)	0.57 (0.17, 1.89)
	Normal weight	1 (reference)	1 (reference)	1 (reference)
	Overweight/Obese	0.83 (0.37, 1.84)	1.23 (0.63, 2.38)	0.80 (0.30, 2.11)
Macrosomia	Underweight	0.24 (0.06, 1.04)	0.28 (0.07, 1.21)	0.23 (0.05, 0.96)*
	Normal weight	1 (reference)	1 (reference)	1 (reference)
	Overweight/Obese	3.08 (1.70, 5.57)***	2.96 (1.71, 5.12)***	3.17 (1.60, 6.25)***
LBW	Underweight	1.03 (0.20, 5.35)	1.11 (0.21, 5.81)	1.14 (0.22, 5.98)
	Normal weight	1 (reference)	1 (reference)	1 (reference)
	Overweight/Obese	1.50 (0.40, 5.59)	1.83 (0.57, 5.84)	2.93 (0.73, 11.83)
LGA	Underweight	0.40 (0.16, 1.02)	0.44 (0.17, 1.12)	0.36 (0.14, 0.93)*
	Normal weight	1 (reference)	1 (reference)	1 (reference)
	Overweight/Obese	2.76 (1.75, 4.37)***	2.58 (1.68, 3.96)***	2.52 (1.50, 4.24)***
SGA	Underweight	2.08 (1.17, 3.70)*	2.04 (1.14, 3.66)*	2.10 (1.18, 3.72)*
	Normal weight	1 (reference)	1 (reference)	1 (reference)
	Overweight/Obese	0.97 (0.52, 1.80)	0.91 (0.52, 1.59)	1.03 (0.50, 2.10)

Values are odds ratios (95% confidence intervals), *p < 0.05, **p < 0.01, ***p < 0.001.

Adjusted for maternal age, Han ethnicity, primiparity, history of miscarriage or abortion, maternal educational level, participant and partner’s income, maternal occupation status, gestational age at delivery, new born sex and gestational weight gain (continuous). GDM, PROM, C-section and PTB was not adjusted for gestational age at delivery and new born sex.

*BMI* body mass index, *OR* Odds ratios, *CI* confidence intervals, *GDM* gestational diabetes mellitus, *PROM* premature rupture of membrane, *C-section* cesarean section, *PTB* preterm birth, *LBW* low birth weight, *LGA* large for gestational age, *SGA* small for gestational age.

### Comparison of Chinese and WHO Asian BMI classifications

Three BMI classifications (the Chinese, WHO Asian and WHO European BMI categories) all predicted increased risk of adverse pregnancy outcomes (Table 2). An exploratory analysis was conducted to identify the BMI cut-off at which the risk of adverse pregnancy outcomes increases, however, there was insufficient power (data not shown).

### Associations between infant neurocognitive development and early pregnancy BMI by different BMI classifications

At around 12 months of age, there were no differences in MDI or PDI scores between BMI subgroups based on the Chinese, WHO Asian and the WHO European BMI classification were used (Table 3). Similarly, there were no associations between BMI subgroup and developmental scores identified by regression analysis (Table 4). No differences in mothers’ and infants’ characteristics were observed between the included and excluded groups for the analysis of child neurodevelopment (Supplementary Table 1).

**Table 3:** Bayley scores compared among the four BMI groups based on the Chinese, WHO Asian or WHO European BMI classifications.

BSID scores	Maternal body mass index (BMI)			*P* value
	Underweight	Normal weight	Overweight/obese	
**Chinese BMI category**	n = 98	n = 736	n = 183	
MDI (mean ± SD)	95.71 ± 15.07	94.37 ± 17.74	94.98 ± 18.58	0.82
PDI (mean ± SD)	86.77 ± 13.08	87.29 ± 14.61	86.16 ± 15.39	0.74
**WHO Asian BMI category**	n = 98	n = 648	n = 271	
MDI (mean ± SD)	95.71 ± 15.07	94.12 ± 17.65	95.38 ± 18.48	0.63
PDI (mean ± SD)	86.77 ± 13.08	87.12 ± 14.64	86.93 ± 15.09	0.98
**WHO European BMI category**	n = 98	n = 791	n = 128	
MDI (mean ± SD)	95.71 ± 15.07	94.13 ± 17.98	96.76 ± 17.29	0.38
PDI (mean ± SD)	86.77 ± 13.08	87.19 ± 14.53	86.29 ± 16.19	0.86

Values are mean ± SD.

**Table 4:** Association (*β* coefficient with 95% confidence interval) of maternal BMI according to the Chinese, WHO Asian or WHO European BMI classifications with the Mental Development Index (MDI) and the Psychomotor Development Index (PDI).

BSID scores	BMI	*β* (95% *CI*)		
		Chinese BMI category	WHO Asian BMI category	WHO European BMI category
MDI	Underweight	0.26 (-4.74, 5.27)	0.62 (-4.42, 5.66)	0.58 (-4.39, 5.55)
	Normal weight	1 (reference)	1 (reference)	1 (reference)
	Overweight/Obese	0.63 (-3.20, 4.46)	1.69 (-1.71, 5.10)	3.71 (-0.84, 8.26)
PDI	Underweight	-0.47 (-4.68, 3.75)	-0.42 (-4.67, 3.83)	-0.39 (-4.58, 3.81)
	Normal weight	1 (reference)	1 (reference)	1 (reference)
	Overweight/Obese	-0.51 (-3.74, 2.71)	-0.17 (-3.05, 2.70)	-0.10 (-3.95, 3.74)

Adjusted for maternal age, gestational weight gain (continuous), Han ethnicity, marital status, history of miscarriage or abortion, smoking or drinking during pregnancy, maternal educational level, participant and partner’s income, gestational age at delivery, new born sex, Apgar score at 1 min, Apgar score at 5 min and child age at neurocognitive follow-up (months).

*BMI* body mass index, *CI* confidence intervals, *MDI* Mental Development Index, *PDI* Psychomotor Development Index.

## Discussion

Aberrant maternal BMI at 11^+0^–14^+6^ weeks of gestation was associated with adverse pregnancy outcomes such as GDM, C-section, macrosomia, LGA and SGA. These results did not depend on whether the Chinese, WHO Asian or the WHO European BMI classification were used. No significant relationship was observed between early pregnancy BMI and infant mental/cognitive or psychomotor development.

The distribution of maternal BMI in our study was similar to that found in most other Chinese populations^2–4^, although the sample size was small. BMI cut-off points for overweight/obesity are important as they enable health care providers to identify high-risk individuals for screening and absolute risk assessment^5^. WHO showed that the BMI cut-off point for observed risk in different Asian populations varies from 22 kg/m^2^ to 25 kg/m^2^^5^ and a study in Hong Kong reported an increased risk of adverse pregnancy outcomes when the maternal BMI was ≥ 23 kg/m^2^^21^. Increased risk of adverse pregnancy outcomes was identified whether overweight was defined as ≥ 23 (WHO Asian) or ≥ 24 kg/m^2^ (Chinese BMI)^5,22^, which are cut-offs derived from non-pregnant populations. Our prospective study confirms that these three cut-off points are useful to apply to a pregnant Chinese population. However, this study lacked power to determine which one is more appropriate. A recent retrospective analysis of 67,248 pregnant women in Hong Kong showed that a BMI ≥ 23 was associated with increased adverse pregnancy outcomes^21^ but this cut-off was not compared to ≥ 24. Thus, there remains a need for large population-based pregnancy studies to determine the most discriminatory BMI cut-offs to use in pregnancy.

Several studies have reported that maternal overweight and obesity plays an important role in the development of GDM^2,3^, which can lead to insulin resistance and systemic inflammation^8^. We also found the higher risk of GDM in women who are overweight/obese. Overweight/obese was associated with an increased risk of C-section in this study based on the Chinese and WHO Asian BMI classification, which was in accord with the findings of previous studies^2,3^. The association between obese and increased risk of C-section may be related to excess maternal pelvic soft tissue which narrows the diameters of the birth canal, decreased rates of cervical dilation and subsequent increased rate of inductions after labour^7^. In addition, the presence of comorbid conditions such as diabetes mellitus or fetal macrosomia, may have influenced decisions about the mode of delivery. We also found that overweight/obese increased the risk of macrosomia and LGA, and that underweight increased the risk of SGA. These findings are consistent with a recent meta-analysis of over 1.6 million Chinese pregnancies that utilised three different standards (the Chinese, WHO Asian and the WHO European BMI classification)^6^. The potential mechanisms linking overweight or obesity and an elevated risk of macrosomia and LGA may be related to insulin resistance, which can lead to increased availability of nutrients to the fetus and thus fetal growth acceleration^23,24^. Underweight mothers are at higher risk of fetal growth restriction because of a smaller plasma volume and lower cardiac output^25^. Additionally, a hypothesis involving DNA methylation at different sites in offspring umbilical cord blood was proposed to explain maternal obesity and underweight effects on neonatal birthweight^26^.

We did not observe any associations between early pregnancy BMI and infant neurocognitive development. Systematic reviews of the influences of maternal pre-pregnancy overweight and obesity on children’s neurocognitive development have reported differing results with negative, mixed, null or positive associations between maternal obesity, and childhood cognitive and physical development of children^18–20^. These various findings may be due to the use of several different methods for measuring or categorizing children’s neurocognitive development related aspects. For example, in a meta-analysis, Adane et al*.*^18^ used children’s physical, cognitive or language developmental scores as tested by the Bayley Scales of Infant Development (BSID) as a measure of children’s neurocognitive development. Sanchez et al*.*^20^ reviewed children’s neurocognitive development including diagnoses of ASD, ADHD, cognitive and intellectual delay and emotional/behavioural problems. Another systematic review and meta-analysis^19^ assessed neurocognitive development by including standardized test scores or curricular-based grades related to specific subject areas, but excluding children with mental disorders that could limit generalizability, such as ADHD, detected delay in communication, adaptive, cognition or socio-emotional domains. Thus, we focus on two major studies that used the BSID test to investigate the association between BMI and children’s neurocognitive development (MDI and PDI scores). These two studies; the Early Childhood Longitudinal Study-Birth Cohort (ECLS-B)^27^, and the INfancia y Medio Ambiente-Environment and Childhood Cohort (INMA) and the mother–child (RHEA) Study^28^ found consistent results for the PDI scores, which did not differ by maternal pre-pregnancy BMI. However, the results for MDI for these two studies were inconsistent. In the ECLS-B cohort study, compared with children of normal weight mothers, MDI scores were lower among children of mothers in all other pre-pregnancy BMI categories^27^. Obesity was associated with a reduction in children’s cognitive development scores in both INMA and RHEA cohorts and overweight was associated with reduced cognitive development scores only in INMA cohort^28^. One possible factor in the null finding of our study was the small number of obese women and the low incidence of obesity in our cohort: 39 (2.9%), 48 (3.6%) and 17 (1.3%) of the women were classified as obese based on the Chinese, WHO Asian and the WHO European BMI cut-off points, respectively, as compared to 1000 (14.0%) in ECLS-B^27^, 150 (7.6%) in INMA and 44 (10.7%) in the RHEA cohorts^28^. Thus, our study had limited power to detect differences in cognitive development of infant by maternal BMI. We analysed both obese and overweight pregnancies together due to the small sample size, and this may have precluded us from detecting any detrimental impact of maternal obesity compared with overweight.

Our study had limitations. Firstly, study participants were not from a nationally representative sample; our study was of women in Chongqing and our sample size was limited and relatively small. The small number of obese women in our study (Table 1), limited the power to assess several outcomes. Therefore, we combined the overweight and obese group as one group, and this may have limited our ability to detect any detrimental impact of maternal obesity compared with overweight. Future studies should consider validating our findings using a larger sample size as well as in rural populations to further understand the association between early pregnancy BMI and pregnancy outcomes and infant neurocognitive development in Chongqing. Secondly, the large loss to follow-up at 12 months may have reduced the power to detect differences in neurocognitive outcomes. That said, the sample size was adequate to demonstrate statistically significant differences in adverse pregnancy outcomes such as GDM.

In conclusion, our study indicated that maternal underweight and overweight/obese identified in early pregnancy are associated with increased risks of various pregnancy complications but are not associated with abnormal infant neurocognitive development. Our findings suggest that health care providers should encourage women to enter pregnancy with a normal BMI.

## Methods

### Ethics approval and informed consent

Ethical approval for this study was provided by the Ethics Committee of Chongqing Medical University, Chongqing, China, No. 2014034. The study was conducted in accordance with the principles in the Declaration of Helsinki 1964 and the International Conference on Harmonisation Good Clinical Practice E6 (ICH-GCP), and in accordance with all applicable regulatory requirements. All patients provided written informed consent prior to participation in the study protocol, including a paternal consent requirement for involving children in research. This trial was registered with the Chinese Clinical Trial Register (ChiCTR-IOR-16007700). The detailed protocol for this study has been published previously^29^.

### Study participants

The study was conducted on pregnant women enrolled in the CLIMB study, a three-armed randomized controlled trial of a complex milk lipid supplement during pregnancy that was first established in Chongqing from September 2015^29^. Women were recruited in their first trimester of pregnancy from the First Affiliated Hospital of Chongqing Medical University and Chongqing Health Centre for Women and Children (CHCWC) in China. Recruitment was completed in June 2017. Eligible gravidas were aged 20–40 years and had a singleton pregnancy. Women with a history of premature delivery before 32 weeks of gestation, known milk allergy or aversion, or lactose intolerance were excluded. Women who withdrew from the study (n = 146), whose pregnancies were terminated (n = 29), who miscarried (n = 12), or were lost to follow up (n = 40), were excluded from the analysis. 1273 women were thus included in the pregnancy and neonatal outcomes analysis. Subsequently, at the 1 year follow up, 1017 children were included in the child neurodevelopment analysis (lost to follow up, n = 256).

Maternal demographic characteristics including age, obstetric history and socioeconomic status were self-reported at the first visit (11—14 weeks of gestation), and early pregnancy weight and height were measured at two hospitals. Gestational age was identified by the date of the last menstrual period, and confirmed by B-ultrasound. Delivery and new born data were obtained via medical records. Maternal independent variables were: early pregnancy BMI categorized using the Chinese, WHO Asian and the WHO European BMI cut-off points^5,22^, calculated using the weight and height measured at the first visit (Table 5).

**Table 5:** Classifications of Body mass index (BMI).

	Chinese BMI category criteria	WHO Asian BMI category criteria	WHO European BMI category criteria
Underweight	< 18.5	< 18.5	< 18.5
Normal weight	18.5–23.9	18.5–22.9	18.5–25.0
Overweight	24.0–27.9	23.0–27.5	25.0–30.0
obese	≥ 28.0	≥ 27.5	≥ 30.0

*BMI* body mass index.

### Pregnancy outcomes

Pregnancy outcomes were diagnosed by experienced obstetricians and abstracted from the medical records. Pregnancy outcomes included: GDM, premature rupture of membrane (PROM), C-section, preterm birth (PTB), macrosomia, low birth weight (LBW), LGA, SGA. The diagnosis of GDM was determined if participants had a 75 g oral glucose tolerance (OGTT) test at 24–28 weeks and met any of the following: fasting plasma glucose ≥ 5.1 mmol/l or 1-h plasma glucose ≥ 10.0 mmol/l or 2-h plasma glucose ≥ 8.5 mmol/l. PROM was defined as rupture of membranes before the onset of labour. PTB was diagnosed based on delivery before 37 weeks. Macrosomia was defined as > 4000 g at delivery and LBW as < 2500 g. LGA and SGA were indicated by birth weight greater than and less than the 90th and 10th percentile for the gestational age by sex, respectively, according to our pervious study^30^.

### Assessment of infant neurocognitive development

The Chinese version of Bayley Scales of Infant Development (BSID) were used to assess mental and psychomotor development for infants who were 2–30 months old^31,32^. The test takes into consideration each infant’s age in days; infants were assessed at around 12 months (range from 11 months and 15 days to 12 months and 15 days) by a trained examiner, with ages corrected for preterm birth. These scales have been formally adapted to the Chinese language and locally standardized to become culturally appropriate, with two main indexes: the Mental Development Index (MDI) and the Psychomotor Development Index (PDI). The test provided raw scores for mental and psychomotor development that were converted into standardized index scores of MDI and PDI (which is using the Mental and Psychomotor raw scores, standardized by age of the child in days at the time of the test examination^33^), based on norms for the Chinese population. These index scores have a mean of 100, and a standard deviation of 15, with a lower score reflecting poorer performance. If an infant refused to cooperate with the examiners to finish the task, a second assessment was arranged within two weeks. If the infant could not cooperate at the second BSID assessment, their data were classified as missing. Quality control checks were conducted by reviewing videotaped evaluations.

### Statistical analysis

All data were analysed using SPSS 19.0 (IBM, USA). Data were described as mean ± SD or median (IQR) for continuous variables, or as percentage for categorical variables. Comparisons of continuous variables were conducted using analysis of variance (ANOVA) or in cases of non-normally distributed variables, non-parametric rank-sum test. For adverse pregnancy outcomes, multivariable logistic regression models were conducted to estimate odds ratios (*OR*) and their 95% confidence intervals (*CI*) across categories of BMI (due to the small samples, we analysed both obese and overweight women together). In the multivariable logistic model, there were adjustments for maternal age, Han ethnicity, primiparity, history of miscarriage or abortion, maternal educational level, participant and partner’s income, maternal occupation status, gestational age at delivery, new born sex and gestational weight gain (GWG). GDM, PROM, C-section and PTB were not adjusted for gestational age at delivery and new born sex. Women with a normal BMI were used as the reference group for other BMI classifications. For mental and psychomotor development, multiple linear regression models were fitted to estimate *β* coefficient and their 95% *CI*s, adjusting for maternal age, Han ethnicity, marital status, history of abortion, smoking or drinking during pregnancy, maternal educational level, participant and partner’s income, gestational age at delivery, new born sex, gestational weight gain (GWG), Apgar score at 1 min and Apgar score at 5 min. Models were checked for collinearity between the variables.

## Supplementary Information

Supplementary Information 1.

## Data Availability

The datasets generated and/or analysed during the current study are available from the corresponding author on reasonable request.
