# Supplementary material for: Associations of early pregnancy BMI with adverse pregnancy outcomes and infant neurocognitive development
Source: Sci Rep. 2021 Feb 15;11:3793. doi: 10.1038/s41598-021-83430-7 (PMC7884778; doi:10.1038/s41598-021-83430-7)
Supplement: Supplementary file 1 — Supplementary Information 1. [file 41598_2021_83430_MOESM1_ESM.docx]

**Supplementary Table 1. C****haracteristics of participants included and excluded from the analysis of** **child neurodevelopment.**

| **Characteristic** | **Included group**  **(n=1017)** | **Excluded group (n=256)** | ***P* value** |
| --- | --- | --- | --- |
| **Maternal characteristics** |  |  |  |
| Maternal age (years, median (IQR)) | 28 (26, 31) | 28 (26, 30) | 0.17 |
| GWG (kg, mean ± SD)^a^ | 8.8 ± 3.6 | 9.1 ± 3.4 | 0.27 |
| Han ethnicity (%) |  |  | 0.44 |
| Yes | 97.8% | 96.9% |  |
| No | 2.2% | 3.1% |  |
| Marital status (%) |  |  | 0.20 |
| Yes | 98.8% | 97.1% |  |
| No | 1.2% | 2.3% |  |
| Primiparity (%) |  |  |  |
| Yes | 79.4% | 76.4% | 0.31 |
| No | 20.6% | 23.6% |  |
| History of miscarriage or abortion (%) |  |  | 0.37 |
| Yes | 47.5% | 44.2% |  |
| No | 52.5% | 55.8% |  |
| Smoking or drinking during pregnancy (%) |  |  | 0.31 |
| Yes | 0.3% | 0.8% |  |
| No | 99.7% | 99.2% |  |
| Chinese BMI category (%) |  |  | 0.11 |
| Underweight | 9.6% | 14.0% |  |
| Normal weight | 72.3% | 71.3% |  |
| Overweight/Obese | 18.0% | 14.7% |  |
| Asian BMI category (%) |  |  | 0.92 |
| Underweight | 9.6% | 14.0% |  |
| Normal weight | 63.8% | 64.1% |  |
| Overweight/Obese | 26.6% | 22.1% |  |
| WHO BMI category (%) |  |  | 0.83 |
| Underweight | 9.6% | 14.0% |  |
| Normal weight | 77.8% | 76.7% |  |
| Overweight/Obese | 12.6% | 9.3% |  |
| **Neonatal characteristics** |  |  |  |
| Gestational age at delivery (week, mean ± SD) | 39.4 ± 1.4 | 39.4 ± 2.0 | 0.75 |
| Birth weight (g, mean ± SD)^b^ | 3315.5 ± 443.7 | 3315.1 ± 464.3 | 0.99 |
| Birth length (cm, mean ± SD)^c^ | 49.8 ± 1.7 | 49.9 ± 2.1 | 0.43 |
| Apgar score at 1 min (median (IQR)) | 10 (9, 10) | 10 (9, 10) | 0.15 |
| Apgar score at 5 min (median (IQR)) | 10 (10, 10) | 10 (10, 10) | 0.77 |
| New born sex (n, %)^d^ |  |  | 0.93 |
| Male | 52.6% | 47.4% |  |
| Female | 52.9% | 47.1% |  |

Abbreviations: BMI: body mass index, GWG: gestational weight gain

Data are mean ± SD, median (IQR) or n (%), **p* < 0.05. *P* values based on χ2 or Fisher’s exact test were used for pairwise comparisons of proportions, T test or Mann-Whitney U test was used for comparisons of continuous variables between the included and excluded groups on the child neurodevelopment

Missing data: ^a^77 last maternal weight, ^b^11 birth weight, ^c^30 birth length, ^d^2 new born sex
